# Interventions for drug-using offenders with co-occurring mental health problems: a systematic review and economic appraisal

**DOI:** 10.1186/s40352-016-0041-y

**Published:** 2016-09-13

**Authors:** Rebecca Woodhouse, Matthew Neilson, Marrissa Martyn-St James, Julie Glanville, Catherine Hewitt, Amanda E. Perry

**Affiliations:** 1 Department of Health Sciences, University of York, ARRC Building Second Floor, Heslington York, YO10 5DD UK; 2 University of Leeds, Leeds, UK; 3 University of Sheffield, Sheffield, UK; 4 YHEC, University of York, York, UK; 5 Trials Unit, University of York, York, UK

**Keywords:** Systematic review, Mental health, Offenders, Drug use

## Abstract

**Background:**

Drug-using offenders with co-occurring mental health problems are common in the criminal justice system. A combination of drug use and mental health problems makes people more likely to be arrested for criminal involvement after release compared to offenders without a mental health problem. Previous research has evaluated interventions aimed broadly at those with a drug problem but rarely with drug use and mental health problems. This systematic review considers the effectiveness of interventions for drug-using offenders with co-occurring mental health problems.

**Methods:**

We searched 14 electronic bibliographic databases up to May 2014 and five Internet resources. The review included randomised controlled trials designed to reduce, eliminate, or prevent relapse of drug use and/or criminal activity. Data were reported on drug and crime outcomes, the identification of mental health problems, diagnoses and resource information using the Drummond checklist. The systematic review used standard methodological procedures as prescribed by the Cochrane collaboration.

**Results:**

Eight trials with 2058 participants met the inclusion criteria. These evaluated: case management (RR, 1.05, 95 % CI 0.90 to 1.22, 235 participants), motivational interviewing and cognitive skills, (MD-7.42, 95 % CI-0.20.12 to 5.28, 162 participants) and interpersonal psychotherapy (RR 0.67, 95 % CI 0.3 to 1.5, 38 participants). None of these trials reported significant reductions in self-report drug misuse or crime. Four trials evaluating differing therapeutic community models showed reductions in re-incarceration (RR 0.28, 95 % CI 0.13 to 0.63, 139 participants) but not re-arrest (RR 1.65, 95 % CI 0.83 to 3.28, 370 participants) or self-report drug use (RR 0.73, 95 % CI 0.53 to 1.01, 370 participants). Mental health problems were identified across the eight trials and 17 different diagnoses were described. Two trials reported some resource information suggesting a cost-beneficial saving when comparing therapeutic communities to a prison alternative.

**Conclusions:**

Overall, the studies showed a high degree of variation, warranting a degree of caution in the interpretation of the magnitude of effect and direction of benefit for treatment outcomes. Specifically, tailored interventions are required to assess the effectiveness of interventions for drug-using offenders with co-occurring mental health problems.

## Background

This systematic review stems from a previous Cochrane review which evaluated the effectiveness of interventions for drug using offenders (anonymised author and web link). The original Cochrane review was updated to produce three further reviews which explored the effectiveness of interventions aimed at reducing drug use and criminal activity in, i) drug using female offenders (Perry et al. 2015b), ii) drug using offenders with co-occurring mental health problems (Perry et al. 2015a), iii) pharmacological interventions (Perry et al. 2015c) and iv) non-pharmacological interventions (Perry et al. in press). This paper summarises the review findings of offenders with co-occurring mental health problems and in addition reports on mental health outcomes and diagnoses.

## Background literature

Mental health issues in offenders are common with over half (64 %) of jail inmates in the US reporting a serious mental health problem (Cosden et al. 2003; Glaze and James 2006; Johnson and Zlotnick 2012; Stein et al. 2011). Such problems are more apparent in women (31 %) than men (14.5 %). A systematic review of 62 surveys found that prisoners were several times more likely to have a diagnoses of psychosis or major depression and ten times more likely to have an antisocial personality disorder than the general population (Fazel and Danesh 2002).

The provision of mental health care in US jails was found to be poor with most providing only intake screening, mental health evaluations and suicidal prevention services (Steadman and Veysey 1997). In addition, the evidence suggests that people who suffer from a mental health problems are disproportionately more likely to be arrested when compared with offenders without mental health problems (Lamb and Weinberger 1998; Lovell et al. 2002). Reasons for this include limited support in the community, poor co-ordination of services and treatment on release, problems accessing treatment, and police and societal attitudes (Cloyes et al. 2010).

Large numbers of offenders also suffer from substance misuse problems and have been consistently reported as a major contributing factor in the increasing population of women offenders (Greenfeld and Snell 1999; Staton-Tindall et al. 2007). The relationship between drugs and crime is also complex. The literature has discussed the issue of whether drug use leads people into criminal activity or whether those who use drugs are already predisposed to such activity. Whilst the majority of women offenders have a history of drug use and drug-related offenses the research evidence suggests that only a small proportion of both men and women receive appropriate treatment and supervision (Taxman 2002).

The combination of drug use and offending behaviour has a substantial economic impact on society and specifically on formal service resources (Byford et al. 2013). In 2011 the National Drug Intelligence Center [NDIC] estimated that the cost to society of drug abuse was $193 billion. Of this they reported that $113 billion was associated with drug related crime, including criminal justice system costs and costs borne by victims of crime. They argue that the cost of treating drug abuse (including health costs, hospitalizations, and government specialty treatment) was a fraction of the overall societal costs. Treatment has also shown to reduce the costs associated with lost productivity, crime, and incarceration across various settings and populations. The largest economic benefit of treatment is seen in avoided costs of crime (incarceration and victimization costs).

Policy initiatives in the US and UK, show a renewed recognition that the criminal justice system (CJS) is not always the best place to manage people suffering from mental health problems. In the case of less serious offenders, several diversionary schemes have been established to provide a mechanism for diverting individuals with mental health problems into treatment programmes instead, or combined with incarceration (Clarke 2010; Sarteschi et al. 2011). Findings from such studies generally show positive improvements on a small number of clinical outcomes. However, the certainty of any causal link is often restricted by type of research design (i.e., quasi-experimental studies), which limit any conclusions about their relative effectiveness (Campbell and Stanley 1963; Cook and Campbell 1979).

Evidence from previous systematic reviews have tended to investigate the effectiveness of interventions for either (i) drug using offenders, or (ii) offenders with mental health problems. Evidence supporting the case for treatment include a range of different treatment options. Some examples include case management, therapeutic community models, cognitive skills and behavioural management and motivational interviewing. Case management evolved to address the needs of prisoner re-entry programmes covering employment, education, health, housing and family support via assessment and connecting clients with the appropriate services (Austin and McClelland 1996). Case management in the US has been applied in Treatment Accountability for Safer Communities (TASC) programmes (Marlowe et al. 2003), and has shown initial effectiveness but without systematic evidence in support of the process.

Similar findings have been found when using cognitive-behavioural approaches. Such programmes tend to include a number of different techniques including self-monitoring, goal setting, self-control training, interpersonal skills training, relapse prevention, group work and lifestyle modification. These have also shown signs of success with offenders in the *general* prison population (Lipsey et al. 2007) but have excluded evaluations of drug-using offenders with co-occurring mental health problems (Andrews et al. 1990; Lipsey et al. 2007; Lipsey and Wilson 1998). Acceptance and commitment therapy (ACT) is an intervention in the form of CBT focussing on an individual accepting personal events rather than attempting to change them and addresses goals for behaviour change. A recent meta-analysis found ACT outperformed control, treatment as usual and waitlist control conditions with individuals with mental health problems in the general population (A-Tjak et al. 2015).

Motivational Interviewing (MI) also has a proven theoretical background showing that such techniques can lead to improved retention in treatment, enhanced motivation to change and reduced offending, (McMurran 2009; Smedslund et al. 2011). Miller and Rollnick (1991) developed MI as a process to motivate change in substance abusers. The technique uses strategies to encourage expressing empathy, avoiding arguing for change and working on ambivalence to strengthen commitment to change. Meta-analyses evidence supports the use of MI as a stand-alone treatment and in combination with more intensive programmes (e.g., Vasilaki et al. (2006). A similar approach used also with people suffering from drug misuse (interpersonal psychotherapy: IPT) problems shows some success in reducing drug misuse with non-criminal justice settings, (Johnson and Zlotnick 2012). IPT and MI are similar in approach with both focussing on building skills to deal with social situations and conflict, as well as additional support for substance use. MI was initially developed for substance abusers with the main focus being client motivation to facilitate change in health-related behaviours (Miller and Rollnick 1991). Whilst IPT was initially developed as a structured therapy for people with depression, with the main focus being the ability to understand psychological symptoms as a response to everyday situations and difficulties (de Mello et al. 2005).

Therapeutic communities (TCs) have been used in the US since the 1960s and more recently in the UK to rehabilitate offenders over a relatively long period of time. The TC ethos focuses on treatment of the ‘whole self’ and not on the drug abuse per se. Residents are instrumental in running the TC and supporting each other through the process and this encompasses the transition between prison and working within the community (e.g., (Mitchell et al. 2012; Prendergast et al. 2011). Evaluations of TC interventions using previous meta-analyses and systematic reviews show modest effects in the reduction of recidivism and drug use in male adult offenders (e.g., (Mitchell et al. (2012); Pearson and Lipton 1999); but have not focused on the co-occurrence between drug misuse and mental health problems and previous evaluations. Therapeutic communities can be tailored to meet the needs of specific groups. Personal reflections is an example of a modified TC treatment, which involves the inclusion of a cognitive behavioural element for individuals with mental health problems and substance use disorders (Sacks et al. 2004).

We found one previous systematic review of 16 randomised controlled trials (RCTs) comprising adult offenders with mental health problems. The findings showed that clozapine was favoured over alternative treatments for improving psychiatric symptoms. The review identified limited evidence to show that discharge planning with benefit application assistance and the use of mental health services on release from incarceration was effective. The authors called for more comparative trials to increase their confidence in the findings (Fontanarosa et al. 2013). Other systematic reviews have evaluated interventions and conducted meta-analyses based on single treatment components (Martin et al. 2012; Morgan et al. 2012). Two reviews focused purely on pharmacological treatments (Griffiths et al. 2012; Huband et al. 2010).

The previous evidence demonstrates varied success with reductions in mental health, crime and drug outcomes but we know little about how interventions for drug using offenders and co-occurring mental health problems can help address treatment and rehabilitation opportunities. For this reason, we believe a systematic evaluation of the existing evidence might help add to the current body of evidence by identifying specific interventions for this group of people. We are also interested in learning more about how such individuals are identified, what diagnoses they are given and how much such interventions might cost. The review was therefore broad and included *any* intervention that was designed to reduce, eliminate or prevent relapse to drug use and/or criminal activity in a sample of participants with drug misuse problems and mental health diagnoses. The review addressed the following questions: (1) Does any treatment for drug-using offenders with co-occurring mental health problems reduce drug use? (2) Does any treatment for drug-using offenders with co-occurring mental health problems reduce criminal activity? (3) How are people identified and diagnosed of a mental health problem? (4) Is there any resource information to enable a cost evaluation?

## Methods

### Search strategy for identification of studies

This review stems from an original Cochrane review (insert anonymised reference). The results of this update meant that we began our searching where the previous review finished (insert anonymous reference). We searched 14 databases and identified records between 2004 and May 2014.^1^ Specified search strategies were developed for each database to include randomised controlled trials (RCTs) of any language. For this purpose, filters retrieved from the Inter TASC Information Specialists’ Sub-Group (ISSG) Search Filter Resource Site (www.york.ac.uk/inst/crd/intertasc/) were used. The following are examples of the search terms used in the searches; prison, offender, substance or drug and reoffend. The search terms and strategies are documented in full in the following publication, (insert anonymised reference).

In addition to the electronic databases, we searched relevant Internet sites (e.g., Home Office, National Institute of Drug Abuse (NIDA)) and we scrutinised the reference lists of all retrieved articles for further references. We undertook catalogue searches of relevant organisations and contacted experts for their knowledge of other published or unpublished studies relevant to the review.

### Study design

The review included RCTs whereby the intervention was designed to reduce, eliminate, or prevent relapse of drug use and/or criminal activity. The comparison arm could contain no treatment, minimal treatment, a waiting list, treatment as usual or another treatment alternative.

### Selection of participants

We included drug-using offenders with co-occurring mental health problems regardless of gender, age or ethnicity. Drug misuse included any study that referred to individuals using occasional drugs or were drug dependent. We defined *offenders* as individuals who were involved in the CJS as residing in special hospitals, prisons, community diversion into court schemes or placed on arrest referral schemes for treatment. Offenders were judged to have a co-occurring mental health problem if the mechanism of identification was explicitly stated in the paper. The mechanism could include one or more of the following methods: i) use of diagnostic gold standard test (e.g., the Diagnostic and Statistical Manual of Mental Disorders, Fourth Edition (DSM-IV)), and/or ii) whereby the nature of the intervention was specifically designed for people with mental health problems (e.g., mental health court), and/ or iii) the participants were described as having a ‘history of psychiatric illness’ or a ‘serious mental disorder’ with co-occurrence of drug use. In some cases, this information was obtained from the participant description and demographic characteristics.

### Types of interventions

The interventions were designed, wholly or in part, to eliminate or prevent relapse to drug-use and/or criminal activity among participants. Based on these criteria we included a number of different psychosocial interventions. Psychosocial interventions have been defined as any intervention that focuses on social or psychological factors rather than biological factors (Ruddy and House 2005).

### Primary outcomes

Two primary outcomes (drug misuse and criminal activity) were reported as dichotomous and continuous outcome measures. Drug use measures were reported as: (1) self-report drug use (including unspecified drug, specific drug use not including alcohol, and Addiction Severity Index Drug Composite Scores); and (2) biological drug use (measured by drug testing by either urine or hair analysis). Criminal activity measures were reported using self-report or official criminal justice records. These included arrest for any offence, drug offences, re-incarceration, convictions, charges and recidivism. Where papers evaluated a number of different follow-up periods, we chose to report the longest follow up period as we felt that such measures provided the most conservative estimate of effectiveness.

### Secondary outcomes

#### Mental health diagnoses and identification

Mental health diagnoses were taken from participant sample descriptions about how people were identified to take part within the trial. For this purpose, we used diagnostic gold standard test evidence, the nature of the intervention and demographic and background characteristics of the persons included.

#### Resource information

Resource information was examined with a full critical appraisal using the (Drummond et al. 1997) checklist. According to Drummond studies containing information on the economics on the intervention are defined as full economic evaluation studies, partial economic evaluation studies, and single effectiveness studies (see Table 1). Full economic evaluations are the comparative analysis of alternative courses of action in terms of both costs (resource use) and consequences (outcomes, effects: (Drummond et al. 1997). This differs from studies which focus solely on costs and resource use, or partial economic evaluations. Studies that use a full economic evaluation do not generally use a single research method; and aim to describe, measure and value all relevant alternative courses of action (e.g. intervention X versus comparator Y), their resource inputs and consequences, are referred to as a Cost-benefit analysis (CBA). Other evaluations which do not take into account all consequences include cost-effectiveness analysis (CEA) and cost-utility analysis (CUA). According to Drummond evaluations need to be comparative as an intervention can only be labelled relative to a benchmark or alternative. Evaluations that are not comparative and do not consider both costs and consequences, and/or a comparator is classified as a partial evaluation (e.g., 1A, 1B, 2). A cost effectiveness or cost study is described if alternatives are compared (e.g., 3A, 3B). However, if only the costs or benefits are described the evaluation is still considered partial evaluation but would be comparative across one-dimension. A study evaluating all aspects of the economic dimensions and a comparative would be considered a full economic costing (e.g. 4).

**Table 1:** Classification scheme for economic evaluations (Drummond 2005)

*Are both costs (inputs) and consequences (outputs) of the alternative examined?*				
*Are two or more alternatives compared?*	No	No		Yes
		Examine consequences only	Examine only costs	
		1B PARTIAL EVALUATION 1B		2 PARTIAL EVALUATION
		Outcome Description	Cost description	Cost-outcome description
	Yes	3A PARTIAL EVALUATION 3B		4 FULL ECONOMIC EVALUATION
		Efficacy effectiveness evaluation (e.g., RCT)	Cost analysis	Cost effectiveness analysis
				Cost Utility analysis
				Cost benefit analysis

*The basis for the assumed risk (e.g. the median control group risk across studies) is provided in footnotes. The corresponding risk (and its 95 % confidence interval) is based on the assumed risk in the comparison group and the relative effect of the intervention (and its 95 % CI)

*CI* confidence interval

GRADE Working Group grades of evidence

**High quality:** Further research is very unlikely to change our confidence in the estimate of effect

**Moderate quality:** Further research is likely to have an important impact on our confidence in the estimate of effect and may change the estimate

**Low quality:** Further research is very likely to have an important impact on our confidence in the estimate of effect and is likely to change the estimate

**Very low quality:** We are very uncertain about the estimate

### Data collection and analysis

The studies were identified using a number of stages. At each stage two reviewers were involved. The stages included i) independently inspecting the search hits by reading the titles and abstracts in a bibliographic database, ii) obtaining a full copy of each potentially relevant study, iii) assessing each study for inclusion, and iv) independently conducting a data extraction and agreement process. In the case of discordance, a third independent reviewer arbitrated. One reviewer undertook all the above stages in the translation of articles that were not written in English. The data extraction was completed using a standardised reporting system and a range of items coded (e.g., information about the study sample, intervention and control group and the key results for our outcome measures). We also grouped the studies by intervention type and setting creating four intervention groups: case management, motivational interviewing and cognitive skills, interpersonal therapy and therapeutic community models.

### Statistical methods

We conducted a narrative presentation of the study details (e.g., author, year of publication and country of study origin), study methods (e.g. random assignment), participants (e.g., number in sample, age, gender, ethnicity, age, mental health status), interventions (e.g., description, duration, intensity and setting), outcomes (e.g., description, follow-up period and reporting mechanism), resource information (e.g., number of staff, intervention delivery, estimated costs and estimated savings) and made notes on the study methodology and quality appraisal information using the Cochrane risk of bias tool.

We were able to standardize our outcome measures using a mean difference (MD) for continuous outcomes measured on the same scale and a standardized mean difference (SMD) for outcomes measured on different scales. Higher scores for continuous measures represented greater harm. We risk ratios (RR) for dichotomous outcomes, and all were presented with 95 % confidence intervals (CIs).

To avoid double counting of our outcome measures (e.g., arrest and parole violation) and follow up time periods (e.g., 12, 18 months) all trials were checked to ensure that multiple studies reporting the same evaluation did not contribute towards multiple estimates of program effectiveness. We followed Cochrane Collaboration guidance and combined intervention and control groups to create single pair wise comparisons. Where this was not appropriate we selected one treatment arm and excluded the others see (Higgins and Green 2011).

### Presentation of study quality and effectiveness

We used Summary of Findings Tables (SoF Table) to provide a concise and transparent summary of the key findings on the quality and certainty of the evidence using GRADE PRO software (Vandvik et al. 2012). The system of assessment was developed by the GRADE Working Group and adopted by the Cochrane Collaboration (Guyatt et al. 2008). The assessment includes an evaluation of the confidence (quality of evidence) and magnitude of effects. A typical table included (i) the primary outcome measures, (ii) a measure of the burden of these outcomes for the control group risk, and the intervention group, (iii) the relative magnitude of effect, (iv) the numbers of participants and studies addressing these outcomes, and (v) a rating of overall confidence in effect estimates for each outcome (Langendam et al. 2013).

The quality of the evidence assesses the extent to which we can be confident that the estimates of effect are correct. These judgements are made using the GRADE system, and are provided for each outcome. The judgements are based on the type of study design (randomised trials versus observational studies), the risk of bias, the consistency of the results across studies, and the precision of the overall estimate across studies. The recommended approach for assessing risk of bias in studies involves the use of a two-part tool that addresses six specific domains, namely, sequence generation and allocation concealment (selection bias), blinding of participants and providers (performance bias), blinding of outcome assessor (detection bias), incomplete outcome data (attrition bias), selective outcome reporting (reporting bias), and other sources of bias. This provides a rating of either Low, Medium or High risk of bias. These ratings use an independent risk of bias score see *Cochrane Handbook for Systematic Reviews of Interventions* (Higgins and Green 2011) and the full report for more details [insert web link to report here].

## Results

**Fig. 1:**
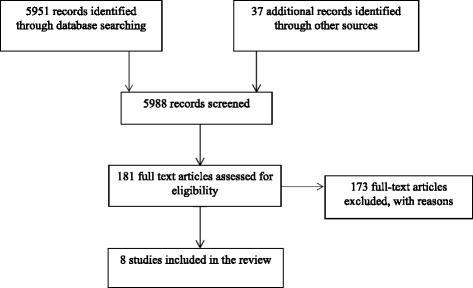
PRISMA Flow Diagram

We identified a total of 5988 records (see Fig. 1). We acquired a total of 181 full-text papers for assessment and excluded 173 papers; identifying eight eligible trials and representing 2058 participants. Of the 173 excluded studies, the main reasons for exclusion were; the study populations were not offenders (*n* = 23), they did not have co-occurring mental health problem (*n* = 41), the intervention was not aimed at reducing drug use or criminal activity (*n* = 7), the study did not report the required drug or crime outcome measures at the pre and/or post-intervention stages (*n* = 36) and the study did not report mental health information (*n* = 16). See the full report for further details (insert anonymised reference),

Seven of the eight trials were conducted in the US; one trial was conducted in Spain. The publications ranged between 1999 and 2014 with a follow up period across the studies between 3 months to 5 years. The treatment length of the interventions (described further in the review) ranged from 8 weeks (Johnson and Zlotnick 2012) to 18 months (Cosden et al. 2003). Seven of the eight comparisons included adult drug-using offenders with a mean age of 30 years. One study investigated the impact of MI with adolescents aged 14 to 19 years (Stein et al. 2011). Three studies included female offenders. In all study populations, the majority of participants were of white ethnic origin.

Four trials represented four different interventions and five publications: (i) court based sentencing with acceptance commitment therapy (ACT) and case management in comparison to treatment as usual (Cosden et al. (2003); (ii) interpersonal psychotherapy in comparison to a psychoeducation course (Johnson and Zlotnick (2012)); (iii) a secure based motivational interviewing programme in comparison to relaxation training (Stein et al. (2011) and, (iv) an evaluation of cognitive behavioural therapy in comparison to acceptance commitment therapy versus a control group (Lanza et al. 2014; Lanza and Gonzalez-Menendez 2013).

The remaining four trials evaluated different models of TC intervention. These included (i) a modified TC in comparison to a combined mental health and substance use education programme (Sacks et al. 2004), (ii) a TC (with voluntary aftercare) in comparison to a waiting list control (Prendergast et al. 2003; Prendergast et al. 2004; Wexler, et al. 1999), (iii) a female adapted TC compared to cognitive behavioural therapy, (Sacks et al. 2008), (iv) a modified TC in comparison to parole supervision with case management (Sacks et al. 2011). Of the eight trials, six were conducted in a ‘secure’ setting and two a ‘court’ setting.

### Case management

#### Criminal activity outcomes

Table 2 reports on one trial of 235 participants for the mental health drug court versus treatment as usual for self-report criminal activity with a mean follow up of 12 months (Cosden et al. 2003). According to the GRADE Working Group, the rating of very low suggests there is uncertainty about the estimate. The assumed risk of the control group is the probability of engaging in criminal activity in the comparison group who have not received the intervention. The RR is slightly higher than 1, indicating a trend towards the intervention group being more likely to engage in criminal behaviour compared to the control group (RR 1.05, 95 % CI 0.90 to 1.22). This single study with a non-significant result should be interpreted with caution.

**Table 2:** Case Management for drug-using offenders with co-occurring mental illness

Outcomes	Illustrative comparative risks* (95 % CI)		Relative effect (95 % CI)	No of Participants (studies)	Quality of the evidence (GRADE)
	Assumed risk	Corresponding risk			
	Control	Mental health court			
Self report dichotomous criminal activity Follow-up: mean 12 months	Study population		RR 1.05 (0.9 to 1.22)	208 (1 study)	⊕⊝⊝⊝ very low
	724 per 1000	761 per 1000 (652 to 884)			
	Moderate				
	725 per 1000	761 per 1000 (652 to 885)			

*The basis for the assumed risk (e.g. the median control group risk across studies) is provided in footnotes. The corresponding risk (and its 95 % confidence interval) is based on the assumed risk in the comparison group and the relative effect of the intervention (and its 95 % CI)

*CI* confidence interval

GRADE Working Group grades of evidence

**High quality:** Further research is very unlikely to change our confidence in the estimate of effect

**Moderate quality:** Further research is likely to have an important impact on our confidence in the estimate of effect and may change the estimate

**Low quality:** Further research is very likely to have an important impact on our confidence in the estimate of effect and is likely to change the estimate

**Very low quality:** We are very uncertain about the estimate

### Motivational interviewing, cognitive skills and relaxation training

#### Drug misuse outcomes

Table 3 shows two outcomes for self-report drug use: continuous and dichotomous. The continuous outcome is represented by one trial (Stein et al. 2011) with 162 participants of low quality as rated by GRADE, for motivational interviewing and cognitive skills for drug using offender’s *vs* relaxation therapy. This quality indicates that further research is likely to impact on the confidence of the estimate and may change the estimate. The study reported no statistically significant reduction in self-report drug use (MD −7.42, 95 % CI −20.12 to 5.28). The dichotomous outcomes were represented by one trial (Lanza and Gonzalez-Menendez 2013) with 41 participants of very low quality as rated by GRADE software, for a mean follow up of three months. The RR is less than 1 indicating a desirable effect of the intervention in reducing drug use (RR 0.92, 95 % CI 0.36 to 2.33).

**Table 3:** Motivational interviewing and cognitive skills for drug-using offenders with co-occurring mental illness

Outcomes	Illustrative comparative risks* (95 % CI)		Relative effect (95 % CI)	No of Participants (studies)	Quality of the evidence (GRADE)
	Assumed risk	Corresponding risk			
	Control	Motivational interviewing and cognitive skills			
Self report drug use continuous Follow-up: mean 3 months	--	The mean self report drug use continuous in the intervention groups was 7.42 lower (20.12 lower to 5.28 higher)	--	162 (1 study)	⊕⊕⊝⊝ low
Self report drug use dichotomous Follow-up: mean 3 months	Study population		RR 0.92 (0.36 to 2.33)	41 (1 study)	⊕⊝⊝⊝ very low
	364 per 1000	335 per 1000 (131 to 847)			
	Moderate				
	364 per 1000	335 per 1000 (131 to 848)			

*The basis for the assumed risk (e.g. the median control group risk across studies) is provided in footnotes. The corresponding risk (and its 95 % confidence interval) is based on the assumed risk in the comparison group and the relative effect of the intervention (and its 95 % CI)

*CI* confidence interval

GRADE Working Group grades of evidence

**High quality:** Further research is very unlikely to change our confidence in the estimate of effect

**Moderate quality:** Further research is likely to have an important impact on our confidence in the estimate of effect and may change the estimate

**Low quality:** Further research is very likely to have an important impact on our confidence in the estimate of effect and is likely to change the estimate

**Very low quality:** We are very uncertain about the estimate

### Interpersonal psychotherapy

#### Drug misuse outcomes

Table 4 reports on one trial (Johnson and Zlotnick 2012) with 38 participants evaluating interpersonal psychotherapy for drug using offenders, for a mean follow up of 3 months. The assumed risk of the control group is the probability of drug use (self-report) occurring in the comparison group who have not received the interpersonal psychotherapy intervention. The RR is less than 1, indicating a desirable effect of having the intervention for reducing drug use (RR 0.67, 95 % CI 0.3 to 1.5).

**Table 4:** Interpersonal psychotherapy for drug-using offenders with co-occurring mental illness

Outcomes	Illustrative comparative risks* (95 % CI)		Relative effect (95 % CI)	No of Participants (studies)	Quality of the evidence (GRADE)
	Assumed risk	Corresponding risk			
	Control	Interpersonal psychotherapy			
Self report drug use dichotomous Follow-up: mean 3 months	Study population		RR 0.67 (0.3 to 1.5)	38 (1 study)	⊕⊝⊝⊝ very low
	474 per 1000	317 per 1000 (142 to 711)			
	Moderate				

*The basis for the assumed risk (e.g. the median control group risk across studies) is provided in footnotes. The corresponding risk (and its 95 % confidence interval) is based on the assumed risk in the comparison group and the relative effect of the intervention (and its 95 % CI)

*CI* confidence interval

GRADE Working Group grades of evidence

**High quality:** Further research is very unlikely to change our confidence in the estimate of effect

**Moderate quality:** Further research is likely to have an important impact on our confidence in the estimate of effect and may change the estimate

**Low quality:** Further research is very likely to have an important impact on our confidence in the estimate of effect and is likely to change the estimate

**Very low quality:** We are very uncertain about the estimate

### Therapeutic community interventions

#### Criminal activity outcomes

Table 5 reports on two criminal activity outcomes: re-arrest and re-incarceration for therapeutic community interventions. Re-arrest is represented by two studies (Sacks et al. 2008; Wexler, et al. 1999) with a total of 798 participants. The outcome is rated as moderate quality of evidence by GRADE software, which suggests further research is likely to impact on the confidence in the estimate of effect. The two studies (Sacks et al. 2008; Wexler, et al. 1999) showed no statistically significant reduction in re-arrest following treatment: (RR 1.65, 95 % CI 0.83 to 3.28, 370 participants) and (RR 0.96, 95 % CI 0.82 to 1.13, 428 participants) respectively. Three trials with re-incarceration outcomes represented 636 participants and are of moderate quality as rated by GRADE software, with a mean follow up of 12 months. Two of these three trials reported a statistically significant reduction in re incarceration at follow up. Sacks et al. (2004) compared a personal reflections TC and voluntary residential aftercare versus mental health programme (RR 0.28, 95 % CI 0.13 to 0.63, 139 participants); and Sacks et al. (2011) compared re-entry modified TC treatment versus parole supervision case management (RR 0.49, 95 % CI 0.27 to 0.89, 127 participants). The third study (Sacks et al. 2008) did not find statistically significant results comparing a TC program versus cognitive behavioural intervention (RR 0.73, 95 % CI 0.45 to 1.19, 370 participants).

**Table 5:** Therapeutic community for drug-using offenders with co-occurring mental illness

Outcomes	Illustrative comparative risks* (95 % CI)		Relative effect (95 % CI)	No of Participants(studies)	Quality of the evidence (GRADE)
	Assumed risk	Corresponding risk			
	Control	Therapeutic community			
Criminal activity - Re-arrests Follow-up: mean 12 months	117/340 (34.4 %)	167/458 (36.5 %)	1st study: 1.65 [0.83, 3.28] 2nd study:0.96 [0.82, 1.13]	798 (2 studies)	⊕⊕⊕⊝ moderate
Criminal activity - Re-incarceration Follow-up: mean 12 months	71/283 (25.1 %)	47/353 (13.3 %)	1st study:0.28 [0.13, 0.63] 2nd study:0.73 [0.45, 1.19] 3rd study:0.49 [0.27, 0.89]	636 (3 studies)	⊕⊕⊕⊝ moderate

*The basis for the assumed risk (e.g. the median control group risk across studies) is provided in footnotes. The corresponding risk (and its 95 % confidence interval) is based on the assumed risk in the comparison group and the relative effect of the intervention (and its 95 % CI)

*CI* confidence interval

GRADE Working Group grades of evidence

**High quality:** Further research is very unlikely to change our confidence in the estimate of effect

**Moderate quality:** Further research is likely to have an important impact on our confidence in the estimate of effect and may change the estimate

**Low quality:** Further research is very likely to have an important impact on our confidence in the estimate of effect and is likely to change the estimate

**Very low quality:** We are very uncertain about the estimate

#### Drug misuse outcomes

Three TC studies reported results for self-report drug use. One study showed a statistically significant reduction: Sacks et al. (2004) (RR 0.58, 95 % CI 0.36 to 0.93, 139 participants); the second study found a near statistically significant reduction: Sacks et al. (2008) (RR 0.73, 95 % CI 0.53 to 1.01, 370 participants); while the third study found no statistically significant reduction: Wexler, et al. (1999) (RR 1.11, 95 % CI 0.82 to 1.49, 576 participants).

### Secondary outcomes

#### Identification and type of mental health problem

Mental health diagnoses varied across the studies with a range of different criteria and assessment mechanisms (see Table 6). The eight trials reported 17 different mental health diagnoses (e.g., depression, post-traumatic stress disorder and generalised anxiety disorder). The most prevalent diagnosis reported in six trials was depression (Johnson and Zlotnick 2012; Lanza and Gonzalez-Menendez 2013; Sacks et al. 2008, 2011; Stein et al. 2011; Wexler, et al. 1999). A range of assessment tools were used to identify the different diagnoses. These included use of the Anxiety Severity Index (ASI), Beck Depression Inventory (BDI) and Mini International Neuropsychiatric Interview (MINI). One study did not specify the criteria used for diagnoses (Wexler, et al. 1999) and one employed a clinical interview and observations by a Psychiatrist (Cosden et al. 2003).

**Table 6:** Mental Health Identification and Diagnoses

Study, year	Criteria used for diagnoses	Description of mental health problem
Cosden et al. 2003	Determined by a psychiatrist/psychologist on the basis of a clinical interview and observations	Mood disorder Schizophrenia Bipolar disorder Other Dual diagnosis
Johnson and Zlotnick 2012	Hamilton Rating Scale for Depression Median duration of index episode in months Number of depressive episodes Number of previous suicide attempts DSM-IV Axis I disorders using the SCID-I/II.	Criteria for a major depressive disorder at least 4 weeks after substance abuse treatment Minimum score of 18 on the Hamilton Rating Scale for Depression.
Lanza and Gonzalez-Menendez 2013	DSM-IV Mini International Neuropsychiatric Interview Anxiety Sensitivity Index	Anxiety Mental health disorders Antisocial personality disorder Major depressive disorder Generalised anxiety disorder
Sacks et al. 2004	DIS	Diagnoses of lifetime Axis I or Axis II mental disorder Antisocial personality disorder
Sacks et al. 2008	Global Severity Index Beck Depression Inventory Lifetime of mental health PTSD Symptom Scale - Interview Posttraumatic Stress Diagnostic Scale	Depression PTSD Lifetime of mental health
Sacks et al. 2011	DSM-IV diagnostic criteria Beck Depression Inventory Post Traumatic Stress Disorder Symptom Scale Brief Symptom Inventory Global Severity Index	Depression PTSD Psychological distress
Stein et al. 2011	CES-D Scale	Scores >16 indicate presence of significant depression. 69.8 % had significant depressive symptoms
Wexler et al. 1999; Prendergast et al. 2003; Prendergast et al. 2004	Not specified	Antisocial personality disorder Phobias PTSD Depression Dysthymia Attention deficit hyperactivity disorder

In addition to the drug and criminal activity outcomes, mental health outcomes were reported across the eight trials. Three trials reported mental health diagnoses at baseline, but did not report any post treatment mental health outcomes (Sacks et al. 2004, 2011; Wexler, et al. 1999). One trial reported some mental health outcomes; but did not provide enough information to enable us to extract the data (Stein et al. 2011).

#### Impact on mental health outcome

Four of the eight trials reported on outcomes of mental health improvement across the intervention and comparison/control groups. Johnson and Zlotnick (2012) reported significantly lower Hamilton Depression Scale scores in the intervention group (interpersonal therapy) compared to the control group. Three studies evaluating a TC model, case management and cognitive behavioural skills showed improvements in scores across both the intervention and control group. The Sacks et al. (2008) study evaluating TC showed improvements in mental health symptoms, assessing using the BDI at 6 months follow up across both intervention groups. This improvement was maintained at 12 months for the TC group, while the control group continued to improve between 6 and 12 month follow up. For case management reductions were reported across both intervention groups for the number of participants meeting criteria for mental health diagnoses (including; mood disorder, schizophrenia, bipolar disorder), determined by a psychiatrist/psychologist at 12 months follow up. For cognitive skills, Lanza and Gonzalez-Menendez (2013) reported reductions across both the intervention and control groups for MINI assessed mental health disorders, including major depressive disorder and generalised anxiety disorder.

### Resource information and economical appraisal

Two trials and seven publications provided some resource information (McCollister et al. 2003; Prendergast et al. 2003; Prendergast et al. 2004; Sacks et al. 2011; Sacks et al. 2004; Sacks et al. 2008; Sacks et al. 2012; Wexler, et al. 1999). The series of Prendergast studies presented economic differences when compared to the one-year TC outcome study. The Prendergast research suggests that optimal cost savings appear to require prison treatment plus aftercare rather than prison treatment alone (McCollister et al. 2003). This was rated as 3B on the Drummond checklist. The series of Sacks TC publications contained some information about cost, but not sufficient to conduct a cost-effectiveness appraisal (Sacks et al. 2004). This was rated on the Drummond checklist as 3A. The authors of this study noted the additional marginal costs (on top of the specific incarceration costs) were $7.37 per day, versus $148.19 cost of a prison day. This suggests a substantial cost saving supporting the use of TC programmes over prison.

## Discussion

### Summary of main results

This systematic review provided evidence from eight trials. The trials were conducted in secure and court settings. We do not have sufficient evidence to be able to suggest whether these interventions work better in one setting as opposed to another. Five different types of treatment interventions were identified. These included: case management, motivational interviewing and cognitive skills, interpersonal psychotherapy and TC models.

### Outcomes of criminal activity and drug use

The TC studies reported statistically significant reductions in subsequent re-incarceration, but not for re-arrest. This finding could be an artefact of the type of outcome. Incarceration (or re-incarceration) to prison takes longer to process and often involves a court case which means that it is likely to be recorded within the time frame of an experimental evaluation. In comparison, ‘arrest’ (or ‘re-arrest’) is more frequent and is recorded in the CJS within a shorter time frame. Sacks et al. (2011) also argues that participation in a treatment option does not necessarily lead to *less* involvement with the CJS, but success might instead be a reduction in the severity of the offence committed such that re-incarceration is less likely. The follow-up studies to the Wexler trial also commented on differential effectiveness of treatment outcomes (Prendergast et al. 2003, 2004; Wexler, et al. 1999). The authors argue that focusing on only one or two outcomes may mask the impact of treatment on other outcome domains that are of interest to various stakeholders. For example, measuring re-arrest or re-incarceration does not reveal the reason for why an individual has returned to correctional supervision. Unanswered questions include (i) the length of time an offender remains in the community until re-arrest, (ii) knowledge about what crimes are committed, and (iii) the reasons for return.

One specifically adapted TC treatment for women offenders compared women assigned to TC treatment or standard treatment, a cognitive behavioural recovery and relapse prevention curriculum referred to in the system as the Intensive Outpatient Program (Sacks et al. 2008). At 6 months the study found that both groups improved significantly on variables of mental health, substance use, criminal behaviour, and HIV risk. The authors note that further exploration of each model for different offender groups is required to permit a precise utility of each model. The authors concluded that the preliminary findings demonstrate the importance of providing gender-specific sensitive and comprehensive approaches within the correctional system (Sacks et al. 2008). The more recent follow-up study investigated outcomes at 6 and 12 months. The outcomes followed a similar pattern, with both groups of women benefiting from treatment. TC treatment was found to be more beneficial than cognitive behavioural therapy at improving re-incarceration rates and lengthening the amount of time spent in the community before subsequent re-incarceration (Sacks et al. 2008).

We noted no statistically significant reductions for criminal activity or self-report drug use with the use of case management via a mental health court; Motivational interviewing with cognitive skills over relaxation training; and Acceptance and Commitment Therapy (ACT) or interpersonal psychotherapy (Cosden et al. 2003; Johnson and Zlotnick 2012; Lanza and Gonzalez-Menendez 2013; Stein et al. 2011). The interpersonal psychotherapy was evaluated using a pilot study of women suffering from major depression and substance use disorder (Johnson and Zlotnick 2012). This feasibility trial was used to assess the applicability of using interpersonal psychotherapy in a prison environment. The findings showed that participants undergoing interpersonal psychotherapy had significantly reduced levels of depression and substance misuse over the attention-matched control (Johnson and Zlotnick 2012).

The study evaluating ACT in comparison to traditional cognitive behavioural therapy found higher levels of abstinence in the ACT (43.8 %) when compared to the control (18.2 %). These findings are similar to other studies that have used ACT albeit in non-incarcerated populations (Hayes et al. 2004). The authors attribute the success of ACT to the nature of the’co-joint’ work between the therapist and client, which aims to increase the flexibility and structure of the therapy allowing the client to have greater autonomy over decision-making. They argue that cognitive behavioural therapy is more systematically directed by the therapist, leaving little scope for responsive change (Lanza et al. 2014; Lanza and Gonzalez-Menendez 2013). In summary, each study represented a singular trial and caution is called for in interpreting the results of the studies as further research is likely to change the impact of confidence.

### Mental health information

In terms of addressing some of the complex issues of individuals with mental health problems and co-occurring substance abuse, the evidence from this systematic review provides starting point for further discussion. Three studies discussed the differential treatment effects on the severity of depression (Cosden et al. 2003; Johnson and Zlotnick 2012; Stein et al. 2011). The Cosden et al. (2003) study noted further understanding of how to help clients with a serious mental health problem with different levels of treatment is needed. The Johnson and Zlotnick (2012) study noted that participants undergoing interpersonal psychotherapy had significantly reduced levels of depression and substance misuse over the attention-matched control. The authors noted that treatment intensity for the individual once released was one key factor to maintaining good outcomes. However, they go on to state that women often experience delays in treatment and service provision on release, and they suggest that alternative service provision such as phone treatment might be helpful in providing a more intensive post-release treatment and useful in times of crisis (Johnson and Zlotnick 2012).

Study descriptions of mental ill health varied (see Table 5). The Cosden et al. (2003) study used a Psychiatrist or Psychologist to conduct a clinical interview to make a mental health diagnosis alongside substance misuse. This resulted in a mental health court sample of individuals diagnosed with a range of mental health problems including mood disorder, schizophrenia, bipolar disorder, and dual diagnosis. Other papers referred to use of the DSM-IV diagnostic criteria (Sacks et al. 2011), but subsequently provided little information with regards to individual mental health needs. Demographic information in the Sacks et al. 2004 study reported on other aspects of mental health prognosis, including lifetime mental health treatment, lifetime in patient care, and prescribed medication (Sacks et al. 2004). The Wexler series of studies reported a range of diagnoses, including antisocial personality disorder, phobias, post-traumatic stress disorder, depression, dysthymia, and attention deficit disorder, but did not describe how these diagnoses were confirmed or assessed within the population.

Six trials reported on change in mental health well-being. Three trials used the Beck Depression Inventory, Global Severity Index, and the Posttraumatic Diagnostic Scale (Sacks et al. 2004, 2008, 2011). Another study reported on Depression but used the Hamilton Rating Scale for Depression (Johnson and Zlotnick 2012). Two studies reported presence of mood disorder alongside schizophrenia, general anxiety disorder, and antisocial personality disorder (Cosden et al. 2003; Lanza et al. 2014; Lanza and Gonzalez-Menendez 2013). Reporting of usable mental health outcomes was limited in the included studies. Three studies reported baseline data but did not provide follow up mental health outcomes (Sacks et al. 2004, 2011; Wexler, et al. 1999) and one study did not provide mental health data in a usable format (Stein et al. 2011). The remaining studies provided mental health data pre and post intervention, however each of the four studies administered a different assessment tool, including BDI, Hamilton Depression Rating Scale, and clinician diagnosed, making it difficult to compare. Future updates of this review will include mental health outcomes in order to assess the impact of treatment on mental health well-being alongside criminality and drug use.

Several successful treatment elements were reported throughout the included trials. First, we noted the issue of treatment engagement as important. In the mental health court trial, informal support from family and friends encouraged the engagement of clients within the community for longer term gains (Cosden et al. 2003). Second, programmes that were specifically adapted to the needs of mental health clients tended to include a cognitive behavioural therapy that emphasised criminal thinking and behaviour alongside psycho-educational classes. The combination of these mechanisms enhanced an individual’s ability to recognise and understand their substance misuse and mental health problems in more detail (Sacks et al. 2004). Third, the longer an individual is engaged in treatment the better the outcome(s) (Wexler, et al. 1999).

### Overall completeness and applicability of evidence

The applicability of the evidence is hindered by the lack of trial coverage to a range of limited treatment options. The limited trials were conducted in the US judicial system, and are therefore, restricted in their generalisation to other CJS outside of the US. The current evidence suggests that TC treatments reduce re-incarceration rates. The review only identifies measures of self-report drug use and must be interpreted with caution. In addition, we can say nothing about whether the treatments are effective in reducing drug use and subsequent criminal behaviour while offenders are on parole or on probation in the community.

### Resource information

Resource and cost information within the studies was evident in two studies, allowing for some comparison of resource information between TC models however both were considered partial evaluations using the Drummond checklist. Regular report of effect sizes would aid calculations for power analysis and provide estimates of the magnitude of treatment effect needed for cost-benefit and cost-effectiveness analysis and would aid decision making for policy makers.

### The trial quality

The available evidence was hindered by the lack of trial coverage to a range of limited treatment options. The trials were conducted in the US judicial system, and are therefore, restricted in their generalisation too other CJS outside of the US. The current evidence suggests that TC treatments reduce re-incarceration rates. The review only identifies measures of self-report drug use and must be interpreted with caution. In addition, we can say nothing about whether the treatments are effective in reducing drug use and subsequent criminal behaviour while offenders are on parole or on probation in the community.

The evidence was often poorly described which prevented the reviewers from making a clear judgement of bias. The imprecision of reporting lowers the quality of evidence, which means that further research is likely to have an impact on our confidence in the estimate of effect. Limitations were described relating to the study design (and leading to problems of selection bias), and in some studies sample sizes were small. The Stein et al. (2011) study was noted as being relatively underpowered and replication of the study is required to enhance the generalisation and external validity of the study findings. Similar modest sample sizes were reported by Sacks et al. (2011); Cosden et al. (2003), who suggested that larger samples should be used to provide a more precise estimate of effect. The Cosden study also reported on the possibility of outcome bias, as the interviewer was not blind to the outcome condition of the participant, and loss to follow-up (25 % of the study sample were lost to follow-up) at 12 months (Cosden et al. (2003).

Another possible selection bias concern in the series of Wexler studies was that participants were randomly assigned to the prison TC treatment and regular prison conditions but not to aftercare (Prendergast et al. 2003, 2004; Wexler, et al. 1999). The authors noted that possible differences in personal motivation may account for some of the positive outcomes associated with participants’ continued support for aftercare services. Subsequently these participants were noted as having the highest’readiness scores’, which suggests that motivation creates an important consideration on client selection (Wexler, et al. 1999).

### Implications for research

We have identified several research implications. First, good-quality research is required to evaluate the effectiveness of interventions in offenders with substance misuse problems and co-occurring mental health problems. Of particular interest are the extended long-term effects of aftercare and the level of contact required with services in the community. Further research to enhance to explore the intensity of different community treatment alternatives following release may help to unravel this process. Second, better descriptions of participants’ mental health problems and more detailed information about mental health diagnoses are required to enable the transferability of information to clinical practice. Such information could also facilitate the use of mental health diagnoses as a moderator within the analysis of the outcomes. Third, trial interventions specifically focusing on females and adolescents are required. In the current review one study contained females, and one study reported on adolescents. Fourth, little is known about the interaction between mental health problems, individual personal characteristics and positive outcomes relating to treatment success. In terms of depression, Stein et al. (2011) attempted to explore some of the differences between participants with few and with many depressive symptoms. Future studies should consider an analysis of existing datasets to reveal which individuals with which mental health diagnosis fair better than others, enabling improved targeting of resources.

Finally, standardising cost and cost-effectiveness information within trial evaluations would help policymakers make decisions about health versus criminal justice costs. New outcome evaluations should consider the length of time to a parolee’s re-arrest or re-incarceration, as this has cost implications. For example, the Prendergast et al. (2003) study found that community residential treatment kept parolees from relapse or recidivism so long as they remained in treatment. Such evaluations provide potential important information for stakeholders and funding bodies involved in distributing budgets to fund such enterprises.

## Conclusions

Two studies employing therapeutic community interventions with aftercare showed a reduction in subsequent re-incarceration. However, the studies generally showed a high degree of variation, warranting a degree of caution in the interpretation of the magnitude of effect and direction of benefit for treatment outcomes. More evaluations are required to assess the effectiveness of interventions for drug-using offenders with co-occurring mental health problems with better mental health descriptions and outcome measurement.

## Footnotes

1 Cochrane Central Register of Controlled Trials (CENTRAL) (1980 to April 2014), MEDLINE (1966 to April 2014), EMBASE (1980 to April 2014), PsycINFO (1978 to April 2014), PASCAL (1973 to November 2004)^a^, SciSearch (Science Citation Index) (1974 to April 2014), Social SciSearch (Social Science Citation Index) (1972 to April 2014), ASSIA (1987 to April 2014), Wilson Applied Science and Technology Abstracts (1983 to October 2004)^a^, Inside Conferences (1993 to November 2004)^a^, Dissertation Abstracts (1961 to October 2004)^a^, NTIS (1964 to April 2014), Sociological Abstracts (1963 to April 2014), HMIC (to April 2014), PAIS (1972 to April 2014), SIGLE (1980 to June 2004)^b^, Criminal Justice Abstracts (1968 to April 2014), LILACS (2004 to April 2014), National Research Register (March 2004)^c^ Current Controlled Trials (December 2009), DrugScope (February 2004) - unable to access, SPECTRA (March 2004)^d^.
